# Myasthenia gravis after glioblastoma resection: paraneoplastic syndrome or coincidence? A unique case report and review of the literature

**DOI:** 10.1007/s00701-021-05035-3

**Published:** 2021-10-29

**Authors:** R. J. Slegers, T. A. M. Bouwens van der Vlis, L. Ackermans, A. Hoeben, A. A. Postma, I. Compter, J. G. J. Hoeijmakers, J. Beckervordersandforth, M. P. G. Broen, O. E. M. G. Schijns

**Affiliations:** 1 Department of Neurosurgery, Maastricht University Medical Center (MUMC+), Maastricht, The Netherlands; 2 Department of Medical Oncology, School for Oncology and Developmental Biology (GROW), Maastricht UMC+, Maastricht, The Netherlands; 3 Department of Radiology, Maastricht University Medical Center (MUMC+), Maastricht, The Netherlands; 4 School for Mental Health and Neuroscience (MHeNS), Maastricht University, Maastricht, The Netherlands; 5 Dept. of Radiation Oncology (Maastro), GROW School for Oncology, Maastricht UMC+, Maastricht, The Netherlands; 6 Department of Neurology, Maastricht UMC+, Maastricht, The Netherlands; 7 Department of Pathology, Maastricht UMC+, Maastricht, The Netherlands; 8 Academic Center for Epileptology, Kempenhaeghe/Maastricht UMC+, Maastricht, The Netherlands

**Keywords:** Myasthenia gravis, Paraneoplastic neurological syndrome, Glioblastoma, Brain tumor, Neuromuscular junction

## Abstract

Paraneoplastic neurological syndromes (PNS) can manifest with every type of malignancy. A well-known syndrome is myasthenia gravis (MG) in combination with thymomas. No association between primary brain tumors and neuromuscular disorders has been described. Here, we present a case of a 65-year-old patient who developed MG, following an uncomplicated, gross-total resection of a glioblastoma. To our knowledge, this is the first case describing the onset of MG during the early postoperative phase after glioblastoma resection. Current criteria of PNS are insufficient when the neurological syndrome is diagnosed at the time of a malignancy or shortly thereafter and should be revisited.

## Introduction

Paraneoplastic neurological syndromes (PNS) are a diverse group of neurological syndromes, with a prevalence of 0.01%, that theoretically can manifest with every type of malignancy [17]. An association has been reported between small-cell lung carcinoma (SLCL) and the Lambert Eaton myasthenic syndrome (LEMS), which occurs in up to 3% of SLCL-patients [5]. A similar syndrome, myasthenia gravis (MG), is associated with thymomas [13]. Both syndromes are autoimmune disorders, of which MG is characterized by antibodies against the acetylcholine receptor (AChR), or similar in function molecules, on the post-synaptic membrane of the neuromuscular junction. This causes a generalized or local weakness of the skeletal muscles. In the majority of cases, ocular muscles are involved, which clinically results in diplopia or ptosis. The incidence of MG is 8–10 new patients per 1,000,000 persons per year with an estimated prevalence of 150 to 250 patients per 1,000,000 persons [3]. Of all MG patients, 10–20% can be considered a PNS associated with a thymoma [13]. Only few cases of possible PNS in combination with primary brain tumors have been described [14]. To the best of our knowledge, this is the first case reporting the onset of MG during the early postoperative phase after glioblastoma (GBM) resection.

## Case presentation

A 65-year-old man with no (autoimmune) medical history was admitted to the emergency room (ER) after an episode with focal motor seizures of the right arm and secondary tonic–clonic generalization. An electroencephalogram (EEG) was performed which showed normal background activity and no signs of epileptic or epileptiform discharges.

**Fig. 1:**
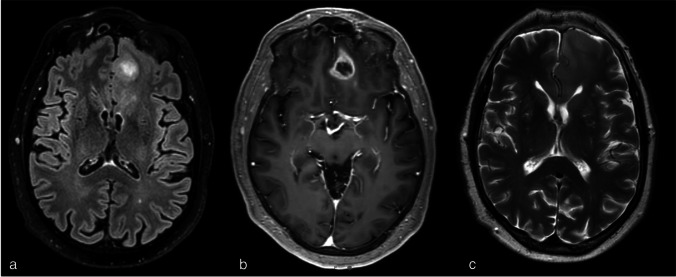
Pre-operative MRI cerebrum: **a** T2-weighted imaging, **b** contrast-enhanced T1-weighted imaging, **c** FLAIR, two (25 mm and 7 mm), rim-enhancing, somewhat erratic delimited lesions left frontal surrounded by an T2/flair hyperintense signal with an edematous aspect of this region, continuing through the genu of the corpus callosum to the right

Magnetic resonance imaging (MRI) (Fig. 1) showed a space-occupying lesion in the left frontal lobe with a larger area of hyperintensity on T2- and flair-weighted images, continuing along the corpus callosum and a smaller area of nodular contrast enhancement with a center of necrosis. The MRI was suggestive for a high-grade glioma. A computed tomography of the thorax and abdomen showed no evidence for a primary tumor and, in retrospect, no thymoma.

After neurosurgical consultation, a partial left fronto-basal lobectomy was performed with gross-total resection of the areas with contrast enhancement. The non-enhancing part of the tumor extending into the corpus collosum remained in situ. The patient recovered well without infections or other complications and was discharged from the hospital on the third postoperative day. Dexamethasone was administered in the postoperative phase and was discontinued 4 days after surgery. The resected tumor specimen showed high cellularity, a high mitosis index, and areas of necrosis surrounded by characteristic pseudo palisading tumor cells. The histological diagnosis was a GBM (WHO grade IV), molecularly characterized by the absence of IDH1/2 mutations, and without MGMT promoter hypermethylation. Adjuvant treatment was planned, according to the *Stupp* treatment protocol: radiotherapy with a total dose of 60 Gy in 30 fractions and concomitant temozolomide 75 mg/m^2^/day for 49 days followed by 6 cycles of temozolomide 150–200 mg/m^2^/day [18].

Before starting adjuvant treatment, the patient presented in the ER with complaints of abnormal sensations in his tongue, together with difficulties in swallowing and chewing. This was two weeks after discharge and 17 days after stopping dexamethasone. In addition, the patient experienced droopy eyelids, which worsened during the day. Several days before presentation, he began having difficulties with keeping his head upright and experienced progressive complaints of shortness of breath. Neurological examination revealed a slight dysarthria, dyspnea on exertion, and bilateral ptosis, increasing with abduction of the eyes > 15 s and at 28-s diplopia.

**Fig. 2:**
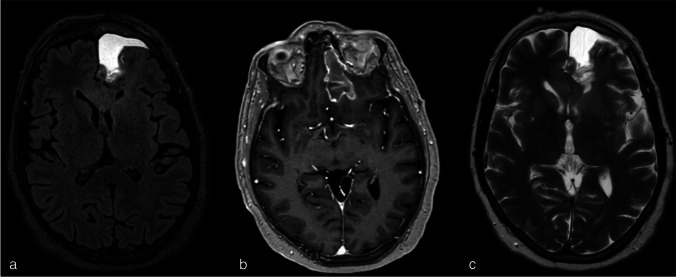
Postoperative MRI cerebrum: **a** T2-weighted imaging, **b** contrast-enhanced T1-weighted imaging, **c** FLAIR

**Fig. 3:**
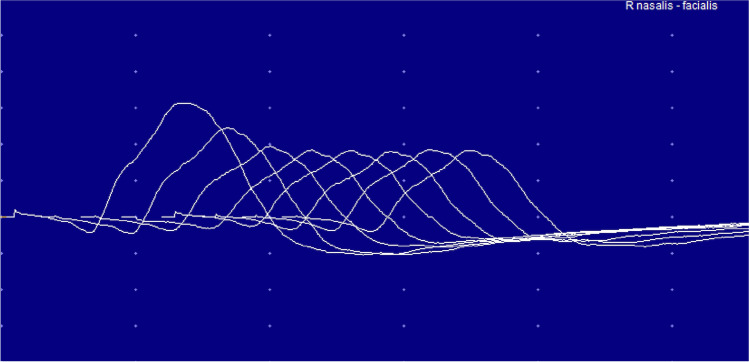
Stimulation of the facial nerve and nasal region with significant decremention of the amplitude with repeated stimulation

A new MRI showed a normal postoperative situation, without residual contrast enhancement and an expected hyperintensity signal on FLAIR images in the corpus collosum (Fig. 2). With clinical suspicion of MG, an electromyogram (EMG) was performed, which showed significant decremention of 30% and 40% respectively on stimulation of the right-sided ulnar and facial nerve, appropriate for a neuromuscular junction disorder (Fig. 3). The patient was prescribed 60 mg prednisone daily and a build-up schedule of pyridostigmine resulting in five times 60 mg daily. Due to the rapid effect of the medication, the patient received local and systemic concomitant and adjuvant treatment for the GBM without delay. The patient returned home without any impairments in daily activities, and a residual ptosis of the right eye. Serology using I-125-alpha-bungarotoxin-labelled AChRs showed AChR antibodies with an auto-antibody level of 0.9 nmol/L (cutoff 0.4 nmol/L) which confirmed the diagnosis of a late-onset MG-type AChR.

Six months after surgery, an MRI scan showed progression of some nodular, contrast-enhanced lesions with limited perfusion ratios, indicating pseudo-progression. Two months after this scan, the patient was admitted at a nearby hospital due to confusion and disorientation. At the ER, 10 mg of dexamethasone was administered. A new MRI showed the known GBM with extensive tumor progression and spread to the contralateral hemisphere with mass effect from the left frontal part. Despite high doses of dexamethasone, frontal and cognitive impairment remained with a Karnofsky Performance Status (KPS) of < 70. Consequently, the patient was transferred from the hospital to start best supportive care. He died 10 months after diagnosis.

## Discussion and conclusions

PNS usually occur as the first sign of a tumor leading to its detection, whereas they are rarely seen during the course or treatment of an oncologic disease. PNS are associated with tumors but are not a direct result of tumor invasion of the adjacent tissue and may be considered an autoimmune condition, set off by proliferating tumor cells, producing proteins that are normally restricted to immune privileged neurons. The immune responses often manifest as anti-neuronal antibodies that can be measured in serum and CSF [8, 9].

In the European database for PNS (*n* = 968), there are no patients with a primary central nervous system malignancy and only very few patients have been described developing an auto-antibody-positive autoimmune encephalitis preceding a glioblastoma [6, 20]. Specifically for MG, two cohorts of 188 and 390 MG patients, reporting a total of 72 extrathymic malignant tumors, respectively, contained only two GBMs after MG onset [2, 12]. The relative underrepresentation of GBM suggest a differential immunological response to CNS oncologic disease when compared to small-cell lung, ovary, or breast cancer [6, 20]. Vice-versa, altered self-proteins (auto-antigens) by specific mutations, misfolding, overexpression, or aberrant degradation before or during tumor formation can elicit an immune response (e.g., p53 and HER2) [1]. The number of tumor-associated antigens has been found to be substantially higher in glioblastoma compared to low-grade gliomas, which also reflects the breach of the blood brain barrier, and therefore, the auto-antibody response [19]. Illustratively, an analysis of peripheral nervous system involvement in a GBM suggests that peripheral electrophysiological aberrations may occur, albeit without the presence of known autoantibodies, suggestive of an induction of humoral autoimmunity [10].

The clinical query in the presented case is whether there is a previously undescribed MG as a paraneoplastic phenomenon of a GBM or whether this concerns an unassociated late-onset MG. The diagnostic criteria for a PNS as set out by Graus et al. [7] distinguish between classical and non-classical syndromes and assume a typical presentation of a PNS before the development of a malignancy. Because MG is not considered in this analysis by Graus et al. [7], the criteria are not sufficient for the current case and otherwise would fit a possible PNS. Furthermore, it is possible that the patient developed MG at an earlier stage, but the disease was masked by the treatment with dexamethasone for the cerebral edema. This could be part of an explanation for the rare occurrence of PNS with a primary brain tumor. It is also known that MG can be provoked by unspecific triggering such as surgery and emotional stress [4].

Some adjuvant treatments for GBM can induce a myasthenic-like syndrome such as tanditinib; however, the presented patient did not receive other therapies than temozolomide according to the Stupp protocol [11, 18].

The poor outcome in the presented case could be due to the extended use of corticosteroids as treatment for MG since this has been linked to poorer overall survival [15]. It is hypothesized that dexamethasone may confer protection from radiotherapy- and chemotherapy-induced genotoxic stress by its antiproliferative effects [16].

Summarizing, we cannot exclude the possibility that the simultaneous occurrence of MG and a GBM is a paraneoplastic phenomenon. However, the current diagnostic criteria do not provide sufficient support for this. The moment of presentation also fits well with typical late-onset AChR MG.

By presenting this case, we hope to increase awareness about a possible correlation between MG and primary brain tumors, although a definite association could not yet be determined and needs further study.
